# Which way is down? Visual and tactile verticality perception in expert dancers and non-experts

**DOI:** 10.1016/j.neuropsychologia.2020.107546

**Published:** 2020-09

**Authors:** Brianna Beck, Alkistis Saramandi, Elisa Raffaella Ferrè, Patrick Haggard

**Affiliations:** a Institute of Cognitive Neuroscience, University College London, 17 Queen Square, London, WC1N 3AZ, United Kingdom; b Department of Psychology, Royal Holloway University of London, Egham, Surrey, TW20 0EX, United Kingdom

**Keywords:** Dance, Gravitational vertical, Proprioceptive, Tactile, Vestibular, Visual

## Abstract

Gravity provides an absolute verticality reference for all spatial perception, allowing us to move within and interact effectively with our world. Bayesian inference models explain verticality perception as a combination of online sensory cues with a prior prediction that the head is usually upright. Until now, these Bayesian models have been formulated for judgements of the perceived orientation of *visual* stimuli. Here, we investigated whether judgements of the verticality of *tactile* stimuli follow a similar pattern of Bayesian perceptual inference. We also explored whether verticality perception is affected by the postural and balance expertise of dancers. We tested both the subjective visual vertical (SVV) and the subjective tactile vertical (STV) in ballet dancers and non-dancers. A robotic arm traced downward-moving visual or tactile stimuli in separate blocks while participants held their head either upright or tilted 30° to their right. Participants reported whether these stimuli deviated to the left (clockwise) or right (anti-clockwise) of the gravitational vertical. Tilting the head biased the SVV away from the longitudinal head axis (the classical E-effect), consistent with a failure to compensate for the vestibulo-ocular counter-roll reflex. On the contrary, tilting the head biased the STV toward the longitudinal head axis (the classical A-effect), consistent with a strong upright head prior. Critically, tilting the head reduced the precision of verticality perception, particularly for ballet dancers’ STV judgements. Head tilt is thought to increase vestibular noise, so ballet dancers seem to be surprisingly susceptible to degradation of vestibular inputs, giving them an inappropriately high weighting in verticality judgements.

## Highlights

• Vestibular, proprioceptive, and external cues contribute to verticality perception.
• The subjective tactile vertical is biased toward the direction of a head tilt.
• The subjective visual vertical is biased away from the direction of a head tilt.
• Ballet dancers are particularly susceptible to vestibular noise caused by tilts.

## 1 Introduction

Perceiving the direction of gravity is vital for balance and orientation in space. The vestibular system is a key source of sensory information about the orientation of one's own body relative to the gravitational vertical. In particular, the otolithic organs within the inner ear detect linear acceleration and head tilts through displacement of hair cells against the otolithic membrane, making them especially important for detecting gravitational forces (Day and Fitzpatrick, 2005). However, other sensory cues also contribute to perception of the body's orientation relative to the gravitational vertical, such as proprioceptive and somatosensory cues to the position of the neck and the trunk (Alberts et al., 2015, 2016; Clemens et al., 2011; Day and Wade, 1969; Groberg et al., 1969; Guerraz et al., 2000; Mittelstaedt, 1997), as well as exteroceptive cues such as the perceived orientation or motion of objects in surrounding space (Bronstein, 1999; Dichgans et al., 1972, 1974; Held et al., 1975; Hughes et al., 1972; MacNeilage et al., 2007; Witkin and Asch, 1948; Zupan and Merfeld, 2003).

According to optimal cue integration models, sensory signals are combined in such a way as to give more weight to precise signals than to noisy signals (Ernst and Banks, 2002; Ernst and Bülthoff, 2004). The precision, or reliability, of a sensory signal could potentially be enhanced through specialised training of that sensory system that reduces its internal noise, and thereby increases the weight given to that sensory modality in multisensory perceptual decisions. With regard to gravity perception, training of the vestibular and/or proprioceptive systems could increase the reliability of those signals and strengthen their contributions to perception of the gravitational vertical. Ballet dancers, for example, exhibit impeccable postural control, having undergone years of intensive training to be able to make precise body movements in space. Studies have demonstrated the superior balance and proprioceptive abilities of professional dancers, compared with amateur dancers or non-dancers (Chatfield et al., 2007; Crotts et al., 1996; Golomer et al., 1999; Jola et al., 2011; Ramsay and Riddoch, 2001; Rein et al., 2011). Those skills may be associated with a greater reliance on vestibular and proprioceptive cues, rather than exteroceptive cues such as vision, to determine the position and orientation of the body (Golomer et al., 1999; Golomer and Dupui, 2000; Jola et al., 2011). Ballet dancers may thus integrate multisensory cues to the gravitational vertical differently than non-dancers do, and that difference could manifest as greater precision and less bias in their verticality judgements.

Previous studies have found that tilting either the body trunk or the head biases perception of the verticality of visual lines (the so-called subjective visual vertical, or SVV). Generally, those studies that employed a high degree of roll tilt (>45–60°) tended to find an Aubert effect (Aubert, 1861), or A-effect, wherein the SVV was biased in the *same* direction as the tilt (Alberts et al., 2015, 2016; Barra et al., 2010; Betts and Curthoys, 1998; Bronstein, 1999; De Vrijer et al., 2008, 2009; Tarnutzer et al., 2009a, 2009b, 2010; Van Beuzekom and Van Gisbergen, 2000). On the other hand, those studies that used smaller roll tilts tended to find a Müller effect (Müller, 1916), or E-effect, wherein the SVV was biased *away* from the direction of tilt (Day and Wade, 1969; Tarnutzer et al., 2009a; Wade, 1968, 1969; Winnick et al., 2019; c.f. Ceyte et al., 2009; Dichgans et al., 1974; Guerraz et al., 1998, 2000). Other studies have explored the subjective haptic vertical (SHV) by asking participants to actively explore a rod with their hands, in the absence of visual input, and judge its orientation relative to the gravitational vertical. Those studies tended to find an E-effect, even at larger roll tilts (Bauermeister et al., 1964; Guerraz et al., 2000; Hazlewood and Singer, 1969; c.f. Fraser et al., 2015).

Inspired by Mittelstaedt's (1983) proposal of an ‘idiotropic vector’ that biases verticality perception toward the longitudinal body axis, several authors (Alberts et al., 2016; Clemens et al., 2011; De Vrijer et al., 2008; 2009) put forward Bayesian inference models of SVV perception to account for the A-effect. For example, Clemens et al. (2011) proposed a Bayesian optimal cue integration model in which somatic graviceptors (Mittelstaedt, 1997) and proprioceptors provide sensory information about the position of the body trunk in space and the position of the head on the trunk, respectively. That information is then combined with direct information about the orientation of the head in space from the vestibular otoliths, as well as a prior prediction that the head is approximately upright, as it is during most of our waking lives. The combination of online proprioceptive, somatosensory, and vestibular signals with an upright head prior yields a perception of the head in space, relative to the direction of gravity. That ‘head-in-space’ percept is then compared with visual information about the location of stimulation on the retina, and with further proprioceptive information about the orientation of the eyes within the head, to produce a SVV judgement. Importantly, vestibular signals are thought to become noisier as the head is tilted, due to the non-uniform distribution of the hair cells on the otoliths (De Vrijer et al., 2008; Tarnutzer et al., 2009b). Therefore, according to this model, large head tilts should paradoxically reduce the weight the brain gives to vestibular information in perception of the gravitational vertical.

Following the model by Clemens et al. (2011), an A-effect (i.e. a bias toward the direction of body/head tilt) would be the inevitable result of combining online sensory information with a prior prediction that the head is upright, but the degree of the A-effect would depend upon the reliability of the vestibular and proprioceptive signals. An E-effect, on the other hand, would be harder to explain. Some have proposed that the E-effect could arise from a vestibulo-ocular counter-roll reflex: when the head tilts to the side, the eyes automatically rotate in the opposite direction to maintain a steady image on the retina. An E-effect might thus indicate a failure of the brain to adequately account for changes in the orientation of the eyes within the head (Alberts et al., 2016; Curthoys, 1996; De Vrijer et al., 2009; Wade and Curthoys, 1997), leading to over-compensation for the head tilt in SVV judgements. If that were the case, however, then we would expect the E-effect to be restricted to situations where visual information is integrated as part of verticality perception. That prediction is not supported by studies of the SHV, which tend to find an E-effect despite the absence of visual input (Bauermeister et al., 1964; Guerraz et al., 2000; Hazlewood and Singer, 1969; c.f. Fraser et al., 2015). However, the SHV is not ideally suited to test our prediction because it employs active, uncontrolled haptic exploration of the stimulus. Such a task involves multiple sensorimotor cues besides tactile inputs, such as efference copies of motor commands (Wolpert and Ghahramani, 2000), proprioceptive signals from the arms and hands, and changing gravitational forces on the upper limbs as they move through space. A task using passive tactile stimulation of the head or the trunk to explore verticality perception (i.e. the subjective tactile vertical, STV) would minimise or eliminate those cues, offering a better test of whether the E-effect extends to judgements of tactile verticality in the absence of visual input.

**Fig. 1:**
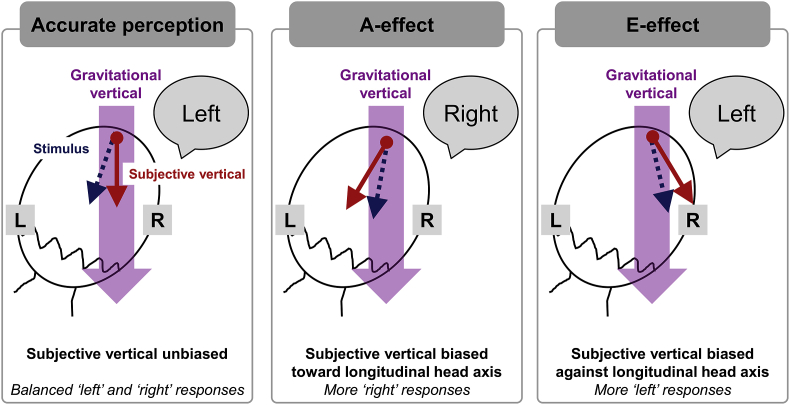
Illustration of potential biases in the subjective visual/tactile vertical during a rightward head tilt. The participant's head is shown from the back. The large purple arrow represents the true gravitational vertical, the solid red arrow represents the participant's subjective perception of vertical, and the dashed blue arrow indicates the downward-moving stimulus applied to the forehead. In the left and middle panels, an example stimulus moves downward and to the left of the gravitational vertical, equivalent to a clockwise rotation of the line traced by the stimulus. A participant who accurately perceives the true vertical will respond ‘left’ (left panel). A participant whose subjective vertical is biased toward the direction of head tilt (an A-effect) will incorrectly respond ‘right’ (middle panel). In the right panel, the stimulus moves downward and to the right of the gravitational vertical, equivalent to an anti-clockwise rotation of the line traced by the stimulus. However, a participant whose subjective vertical is biased away from the direction of head tilt (an E-effect) will incorrectly respond ‘left’ (right panel). (For interpretation of the references to colour in this figure legend, the reader is referred to the Web version of this article.)

Here, we tested the visual and tactile verticality perception of female ballet dancers and non-dancers of similar ages. Participants judged the direction of downward-moving visual stimuli presented in front of their face and equivalent tactile stimuli drawn on their forehead while either holding their head upright or tilted 30° to the right (in a clockwise direction). They judged the direction of these stimuli relative to the gravitational vertical, which either moved downward and to the left (i.e. clockwise with respect to vertical) or downward and to the right (i.e. anti-clockwise with respect to vertical; Fig. 1). We measured both the precision of their judgements and any systematic biases in the subjective visual vertical (SVV) and the subjective tactile vertical (STV). Based on the ocular counter-roll hypothesis (Alberts et al., 2016; Curthoys, 1996; De Vrijer et al., 2009; Wade and Curthoys, 1997) and previous studies using head or body tilts less than 45–60° (Day and Wade, 1969; Tarnutzer et al., 2009a; Wade, 1968, 1969; Winnick et al., 2019), we expected to find an E-effect in the SVV. On the other hand, we expected to find an A-effect in the STV based on the Bayesian inference models of verticality perception with an upright head prior (Alberts et al., 2016; Clemens et al., 2011; De Vrijer et al., 2008; 2009), because the orientation of the eyes in the head would not be relevant in the absence of visual stimulation.

With regard to dance experience, we expected ballet dancers to make less biased verticality judgements than non-dancers, due to their extensive vestibular and proprioceptive training. Since biases arise from tilting the head, the reduced bias would manifest as a smaller difference in the point of subjective verticality (PSV) between upright and tilted head positions in dancers, compared with non-dancers. We also expected dancers to make more precise verticality judgements in the tilted head position, where verticality judgements would be more difficult. We were further interested in exploring whether any advantages of dance expertise might be specific to the stimulation modality (i.e. greater difference between dancers and non-dancers in the tactile modality than the visual modality, or vice versa).

## 2 Material and methods

### 2.1 Participants

A power analysis conducted in G*Power 3.1.5 (Faul et al., 2007), based on a desired power of 0.8 and an average effect size of η_p_^2^ = 0.2 from a series of experiments comparing effects of proprioceptive and vestibular manipulations on the SVV and the SHV (Fraser et al., 2015), indicated a required sample size of approximately 46 participants. We recruited 47 female participants (25 ballet dancers and 22 non-dancers) with normal or corrected-to-normal vision and no history of vestibular or psychiatric disorders (Table 1). Ballet dancers were recruited via e-mails or in-person visits to dance companies in the London area, and were compensated for their participation at a rate of £7.50 per hour. They were eligible to participate if they had completed at least ten years of ballet training (at least one year of which was professional training) and had been training at least five times a week for the past two years. Non-dancers were students recruited from the University College London (UCL) Psychology and Language Sciences research participant database. They received partial course credit in exchange for their participation. All participants gave written informed consent to participate in the study, which was approved by the University College London research ethics committee. All work was carried out in accordance with The Code of Ethics of the World Medical Association (Declaration of Helsinki).

**Table 1:** Demographics of ballet dancers (n = 25) and non-dancers (n = 22).

	Ballet dancers	Non-dancers
Age (years)	23.16 ± 5.53	19.23 ± 1.34
Handedness	21 right, 3 left, 1 ambidextrous	21 right, 1 left, 0 ambidextrous
Physically active?^a^	25 yes, 0 no	4 yes, 18 no
Age at start of ballet training (M ± SD)	5.64 ± 3.76	N/A
Years of ballet practice (M ± SD)	16.68 ± 6.31	N/A
Years of intensive practice (M ± SD)^b^	9.54 ± 6.55	N/A
Years of professional training (M ± SD)	5.66 ± 5.68	N/A
Current dance role	12 professional dancers, 2 teachers, 11 trainees	N/A

a Being physically active was defined as practicing any form of physical activity more than 3 times per week.

b Intensive ballet practice was defined as practicing at least 5 times per week.

### 2.2 Materials and apparatus

A Phantom Premium 1.0 high-precision haptic robotic device (3D Systems, Rock Hill, SC, USA) was used to deliver stimuli on the participant's forehead (in the tactile stimulation condition) or approximately 45 cm in front of their eyes (in the visual stimulus condition). Each stimulus was 2.6 cm long, and the robotic arm moved at a rate of 1.73 cm/s. MATLAB software (Mathworks, Inc., Natick, MA, USA) with the Geomagic Open Haptics Toolkit (3D Systems) and the Prok.Phantom COM.NET component (prok-phantom.googlecode.com) was used to control the device and collect participants' key press responses. Participants placed their head on a chin rest secured to the desk, to ensure that they did not move from the desired position during the experimental blocks. The experimenter used a protractor to monitor the participant's posture and ensure that they remained in the desired position.

**Fig. 2:**
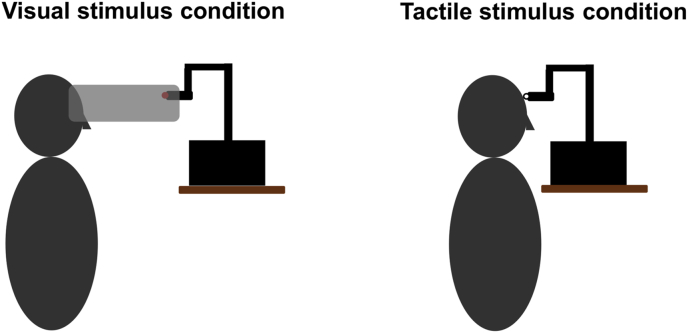
Schematic drawings of the Phantom Premium 1.0 haptic robotic device delivering visual stimulation via a red LED moved in front of the eyes at the end of the black cylinder (left) and tactile stimulation to the forehead via a round pin head (right). Note that the lights in the room were switched off during visual stimulation and the participant was blindfolded during tactile stimulation. (For interpretation of the references to colour in this figure legend, the reader is referred to the Web version of this article.)

To estimate the subjective visual vertical (SVV), a 3-mm diameter red LED was attached to the end of the robotic arm. A black paper cylinder approximately 20 cm in diameter was placed around the participant's face and black fabric was draped over their head to prevent them from seeing any visual cues to verticality (e.g. the corners of the room). The robotic arm was positioned at the other end of the cylinder, about 45 cm in front of the participant's eyes (Fig. 2, left). Additionally, participants were tested in a dark room, and all objects and surfaces within the participant's view were covered in black plastic and/or black tape to ensure that only the red LED was visible.

To estimate the subjective tactile vertical (STV), a 4-mm round pin head was attached to the end of the robotic arm and drawn down the participant's forehead (Fig. 2, right). The participant wore an eye mask to block any visual cues and plastic goggles to protect their eyes from any unintended contact with the tactile stimulus. The robotic arm was positioned so that it delivered light touch to the participant's forehead to minimise friction against the skin.

### 2.3 Procedure

Participants were asked to judge whether lines drawn downward on their forehead or in front of their eyes deviated to the left (clockwise) or the right (anti-clockwise) of the gravitational vertical, defined as the imaginary line that, if drawn straight down from a point in space, would form a 90° angle with the floor (Fig. 1). As a further example, they were told that the gravitational vertical is the direction in which a ball would drop if released from one's hand. They were also shown illustrated examples of ‘left’ and ‘right’ stimuli drawn on paper.

Each participant completed four experimental conditions: Visual stimulus + Upright head, Visual stimulus + Tilted head, Tactile stimulus + Upright head, and Tactile stimulus + Tilted head. Condition order was randomised across participants. In the upright head conditions, participants positioned their head upright on the chin rest. In the tilted head conditions, the experimenter used a protractor to adjust the angle of the chin rest and help participants tilt their head 30° to the right. The participant maintained that position until the end of each block. A head tilt of 30° was chosen because it is a moderate degree of inclination that participants could comfortably maintain for an extended period of time. Only rightward head tilts were tested in this experiment.

Each condition consisted of three blocks of 40 trials each. We used a method of constant stimuli. On each trial, the robotic device delivered a single visual or tactile motion stimulus (2.6 cm long, 1.73 cm/s) that moved downward and angled to the left or right of the gravitational vertical. In the visual condition, the stimulus was situated approximately 45 cm in front of the participant's eyes. At the beginning and the end of each stimulus, the robotic arm remained static for 1 s. Six different angles were used: −25°, −15°, −5°, 5°, 15°, and 25°. Negative values indicate angles to the left of the vertical (clockwise), and positive values indicate angles to the right of the vertical (anti-clockwise). Each stimulus angle was repeated 12 times in a randomised order, and the starting position of the stimulus was jittered on the horizontal axis. A beep at the end of the stimulus indicated that participants should make their response. Using a keypad in their right hand, they pressed one key if the stimulus was angled to the right and another key if it was angled to the left. A single trial lasted approximately 8 s, and the entire experimental session took about 2 h to complete, including the time allocated to instructions, practice blocks (12 trials each for the visual and tactile conditions), and rest breaks between blocks.

### 2.4 Design and analysis

The experiment used a 2 × 2 × 2 (modality x posture x group) mixed-factors design. The two within-subjects factors were stimulus modality (visual or tactile) and head posture (upright or tilted 30° to the right), and there was one between-subjects factor of dance expertise (ballet dancers and non-dancers). The Palamedes Toolbox for MATLAB (Prins and Kingdom, 2018) was used to fit logistic psychometric functions to the data for each participant in each condition using a maximum likelihood criterion, and to estimate the slope as a measure of precision and the point of subjective verticality (PSV) as a measure of bias. The slope is the rate at which the log odds of responding ‘right’ increases as the stimulus angle is deviated toward the right (anti-clockwise). It is inversely related to the standard deviation of the function used to fit the data and thus constitutes a measure of precision (Kingdom and Prins, 2016, p. 22). The PSV is the stimulus angle, derived from the psychometric function, at which the participant is equally likely to respond either ‘right’ or ‘left’ (i.e. the 50% threshold).

## 3 Results

### 3.1 Point of subjective verticality (PSV)

First, we conducted a 2 × 2 × 2 mixed factors analysis of variance (ANOVA) on the PSV values, with dance expertise as a between-subjects factor (ballet dancers vs non-dancers) and stimulus modality (visual vs tactile) and head posture (upright vs tilted) as within-subjects factors. Nine participants (7 dancers and 2 non-dancers) had flat slopes (<0.02) in at least one of the visual conditions (visual-upright and/or visual-tilted), so we were unable to estimate the PSV from their psychometric functions. Those participants were excluded from this analysis.

Negative PSV values indicate that downward deviations to the left of the direction of gravity, from a first-person perspective, are perceived as subjectively vertical. This represents a bias of the PSV in the same clockwise direction as the head tilt (i.e. an A-effect), and thus a tendency to make more 'right' responses (Fig. 1, middle). Conversely, positive PSV values indicate that downward deviations to the right of the direction of gravity are perceived as subjectively vertical. This represents a bias in the anti-clockwise direction, opposite the direction of head tilt (i.e. an E-effect), and thus a tendency to make more 'left' responses (Fig. 1, right).

**Fig. 3:**
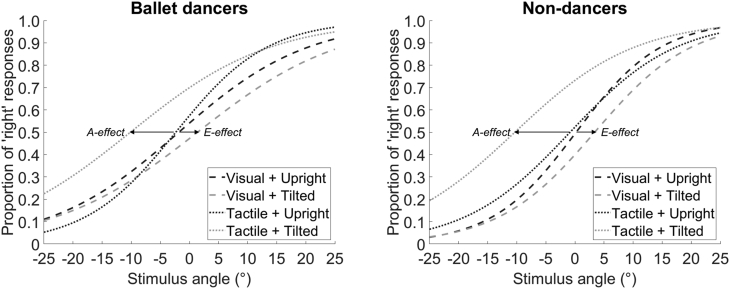
Average psychometric functions showing the effect of tilting the head 30° to the right on verticality judgements of visual (dashed lines) and tactile stimuli (dotted lines). Shifts toward the left indicate an A-effect (i.e. the subjective vertical is biased in a clockwise direction toward the longitudinal head axis), whereas shifts toward the right indicate an E-effect (i.e. the subjective vertical is biased in an anti-clockwise direction away from the longitudinal head axis). Average slope values were calculated from the full participant sample (25 dancers, 22 non-dancers), whereas the average point of subjective verticality (PSV) values (i.e. 50% threshold) were calculated from a smaller sample (18 dancers, 20 non-dancers) excluding those participants with flat slopes in at least one condition.

There was a main effect of stimulus modality, *F*(1, 36) = 40.46, *p* < .001, η_p_^2^ = 0.529, a main effect of head posture, *F*(1, 36) = 7.87, *p* = .008, η_p_^2^ = 0.179, and an interaction between those two factors, *F*(1, 36) = 37.70, *p* < .001, η_p_^2^ = 0.512. Simple main effects tests of posture showed an E-effect in the visual modality, with the PSV biased toward the opposite direction when the head was tilted 30°(M = 2.44°, SD = ±7.13°, 95% CI = [0.47° 4.42°]) relative to when the head was held upright (M = −0.76°, SD = ±5.92°, 95% CI = [-2.73° 1.22°]), *F*(1, 36) = 5.50, *p* = .025. Conversely, there was an A-effect in the tactile modality, with the PSV biased toward the longitudinal head axis when the head was tilted 30° (M = −10.24°, SD = ±6.65°, 95% CI = [-12.22° -8.27°]) relative to when it was held upright (M = −1.55°, SD = ±4.61°, 95% CI = [-3.53° 0.42°]), *F*(1, 36) = 40.16, *p* < .001 (Fig. 3).

There was no main effect of dance expertise on the PSV, *F*(1, 36) = 1.70, *p* = .200, η_p_^2^ = 0.045, nor did dance expertise interact with the other factors (dance expertise x stimulus modality: *F*(1, 36) = 0.41, *p* = .524, η_p_^2^ = 0.011; dance expertise x head posture: *F*(1, 36) = 0.20, *p* = .661, η_p_^2^ = 0.005; dance expertise x stimulus modality x head posture: *F*(1, 36) = 0.19, *p* = .666, η_p_^2^ = 0.005). This shows that both ballet dancers (Fig. 3, left) and non-dancers (Fig. 3, right) experienced similar E-effects in the visual modality and A-effects in the tactile modality.

### 3.2 Percentage of ‘right’ responses

In the preceding PSV analysis, we had to exclude more dancers (n = 7) than non-dancers (n = 2) because the slopes of their visual psychometric functions were too flat to determine the PSV. Those participants were presumably the ones who found the task the most difficult, raising the possibility that removing them may have biased our PSV results. To exclude this possibility, we conducted a 2 × 2 × 2 mixed factors ANOVA with the same between- and within-subjects factors on an alternative measure of bias: the percentage of ‘right’ (vs ‘left’) responses, using the data from all participants (N = 47). Similarly to the PSV analysis, there was a main effect of stimulus modality, *F*(1, 45) = 21.52, *p* < .001, η_p_^2^ = 0.323, a main effect of head posture, *F*(1, 45) = 12.39, *p* = .001, η_p_^2^ = 0.216, and an interaction between those two factors, *F*(1, 45) = 43.57, *p* < .001, η_p_^2^ = 0.492. In the visual condition, tilting the head 30° to the right led participants to make fewer ‘right’ responses (M = 48.4%, SD = ±10.8%, 95% CI = [45.8% 50.9%]) relative to when the head was held upright (M = 51.7%, SD = ±9.0%, 95% CI = [49.1% 54.2%]), *F*(1, 45) = 4.20, *p* = .046. Conversely, in the tactile modality, tilting the head 30° to the right led participants to make more ‘right’ responses (M = 62.7%, SD = ±8.5%, 95% CI = [60.1% 65.3%]) relative to when the head was held upright (M = 50.8%, SD = ±7.0%, 95% CI = [48.2% 53.4%]), *F*(1, 45) = 53.10, *p* < .001. There was no main effect of dance expertise, *F*(1, 45) = 1.75, *p* = .193, η_p_^2^ = 0.037, and dance expertise did not interact with the other factors (dance expertise x stimulus modality: *F*(1, 45) = 2.19, *p* = .146, η_p_^2^ = 0.046; dance expertise x head posture: *F*(1, 45) < 0.01, *p* = .987, η_p_^2^ < 0.001; dance expertise x stimulus modality x head posture: *F*(1, 45) = 1.10, *p* = .300, η_p_^2^ = 0.024). These findings corroborate the PSV analysis, and indicate that removing the 9 participants with flat psychometric functions in at least one condition did not bias our PSV results.

### 3.3 Precision of verticality judgements (slope)

To look at the precision of verticality judgements, we conducted a 2 × 2 × 2 mixed factors ANOVA on the slope values obtained from the psychometric functions. A higher slope indicates more precise (but not necessarily more accurate) judgements.

For the first analysis, we included those participants with flat slopes in some experimental conditions to avoid biasing our results (N = 47). Note that flat slopes might be meaningful and relevant to our hypotheses, particularly where there may be differences between dancers and non-dancers using the same stimuli, because a flat slope indicates minimal sensitivity to stimulus direction. There was a main effect of head posture, *F*(1, 45) = 22.04, *p* < .001, η_p_^2^ = 0.329, indicating that tilting the head reduced the precision of verticality judgements (M = 0.09, SD = ±0.04, 95% CI = [0.08 0.11]) relative to holding the head upright (M = 0.12, SD = ±0.05, 95% CI = [0.10 0.13]). There was also a three-way interaction between head posture, stimulus modality, and dance expertise, *F*(1, 45) = 4.69, *p* = .036, η_p_^2^ = 0.094. Simple main effects tests of posture showed that tilting the head particularly affected the precision of ballet dancers' judgements about the verticality of tactile stimuli, *F*(1, 45) = 24.80, *p* < .001. This can be observed in the dotted lines representing the tactile stimulation conditions in the left-hand panel of Fig. 3; the slope of the logistic curve is much shallower in the dancers' ‘Tactile + Tilted’ condition, compared with their ‘Tactile + Upright’ condition. The effect of posture was not significant in any of the other pairwise, orthogonal contrasts (dancers' visual judgements: *F*(1, 45) = 1.01, *p* = .320; non-dancers’ tactile judgements: *F*(1, 45) = 1.75, *p* = .193; non-dancers’ visual judgements: *F*(1, 45) = 3.22, *p* = .080). There were no main effects of stimulus modality, *F*(1, 45) = 0.05, *p* = .820, η_p_^2^ = 0.001, or dance expertise, *F*(1, 45) = 3.43, *p* = .071, η_p_^2^ = 0.071, and no two-way interactions (head posture x stimulus modality: *F*(1, 45) = 2.82, *p* = .100, η_p_^2^ = 0.059; head posture x dance expertise: *F*(1, 45) = 1.81, *p* = .186, η_p_^2^ = 0.039; stimulus modality x dance expertise: *F*(1, 45) = 3.55, *p* = .066, η_p_^2^ = 0.073).

Although flat psychometric function slopes could indicate a genuine lack of sensitivity to stimulus direction, which would be relevant to our hypotheses, they might also arise from extraneous factors such as a lack of attention to the task. To determine whether any of the effects we found on precision were driven by the inclusion of participants with flat slopes, we repeated the analysis on the precision of verticality judgements after removing the 7 dancers and 2 non-dancers who displayed flat slopes in at least one of the visual conditions. The pattern of results remained the same. There was a main effect of head posture, *F*(1, 36) = 22.01, *p* < .001, η_p_^2^ = 0.379, and a three-way interaction between head posture, stimulus modality, and dance expertise, *F*(1, 36) = 4.65, *p* = .038, η_p_^2^ = 0.114. There were no main effects of stimulus modality, *F*(1, 36) = 3.86, *p* = .057, η_p_^2^ = 0.097, or dance expertise, *F*(1, 36) = 1.88, *p* = .179, η_p_^2^ = 0.050, and no two-way interactions (head posture x stimulus modality: *F*(1, 36) = 2.14, *p* = .152, η_p_^2^ = 0.056; head posture x dance expertise: *F*(1, 36) = 3.28, *p* = .079, η_p_^2^ = 0.083; stimulus modality x dance expertise: *F*(1, 36) = 1.01, *p* = .321, η_p_^2^ = 0.027).

## 4 Discussion

Our study investigated the roles of dance expertise, head posture, and stimulus modality (tactile vs visual) in perception of the direction of gravity. Female ballet dancers and non-dancer control participants judged the angular deviations of downward-moving visual stimuli or tactile stimuli, relative to the gravitational vertical. Because of their extensive proprioceptive and vestibular training, we predicted that the dancers, compared with non-dancers, would be less biased by a tilted head posture, and that their judgements in the tilted head position would be more precise than those of the non-dancers. On the contrary, dancers and non-dancers showed equivalent precision in the upright head conditions, but the dancers were particularly affected by tilting the head: their tactile verticality judgements became less precise. Moreover, both dancers and non-dancers showed similar biases in response to tilting their head 30° to the right. In the visual stimulation condition, they showed an E-effect—their perception of the gravitational vertical was biased *against* the direction of the head tilt. Conversely, in the tactile stimulation condition, they showed an A-effect—their perception of the gravitational vertical was biased *toward* the direction of the head tilt.

Previous studies of the subjective visual vertical (SVV) have tended to show an E-effect with head or body tilts less than 45–60° and an A-effect with greater tilts (Alberts et al., 2015, 2016; Aubert, 1861; Barra et al., 2010; Betts and Curthoys, 1998; Bronstein, 1999; Day and Wade, 1969; De Vrijer et al., 2008; Müller, 1916; Tarnutzer et al., 2009a, 2009b; 2010; Van Beuzekom and Van Gisbergen, 2000; Wade, 1968, 1969; Winnick et al., 2019). Our study used a small rightward head tilt of 30° and found an E-effect on the SVV, consistent with that general trend. However, there is a lack of consistency amongst previous findings, and several studies have found A-effects at smaller inclinations (Ceyte et al., 2009; Dichgans et al., 1974; Guerraz et al., 1998, 2000). Our study alone cannot resolve those contradictions, but methodological differences might offer some explanation. For example, Fraser et al. (2015) suggested that the quality of the visual stimulus could be a key difference; at an intermediate body tilt of 45°, they found an A-effect when using a sharply defined visual line to test the SVV, but an E-effect when using shorter, blurry visual lines. Rather than using a static visual line, we used a single-point LED stimulus that moved downward at an angle, drawing a line in the participant's field of vision. Perceiving the direction of motion of this stimulus requires comparing visual spatial information over time. This kind of dynamic stimulus may therefore be less clear than a static line; indeed, some participants, especially ballet dancers, found it difficult to perceive the visual motion clearly. The indistinctness of our visual stimulus could also have contributed to our finding of an E-effect in the SVV.

Some authors have suggested that an SVV E-effect could arise from the ocular counter-roll reflex (Alberts et al., 2016; Curthoys, 1996; De Vrijer et al., 2009; Wade and Curthoys, 1997). When the head is tilted during visual fixation, the eyes automatically rotate in the opposite direction to provide a stable visual percept of an upright world. Perception of the SVV as rotated away from the direction of head tilt (i.e. an E-effect) could thus arise from a failure of verticality perception to account for the ocular counter-roll reflex (Curthoys, 1996). Although we did not measure ocular counter-roll directly, our results are consistent with this interpretation. Such an effect may have been particularly noticeable in our study, as we went to great pains to eliminate any possible visual cues to the gravitational vertical, leaving only the target stimulus itself visible to participants. Contrary to Clemens et al.'s (2011) Bayesian cue integration model of visual verticality perception, our result suggests that participants fail to integrate ‘eyes-in-head’ cues from the ocular muscles when judging the verticality of visual stimuli in an otherwise visually deprived environment. Alternatively, ‘eyes-in-head’ cues may be noisy and, therefore, overshadowed by a prior prediction that the eyes are upright within the head (De Vrijer et al., 2009). Either way, an E-effect may represent an attempt to compensate for the head tilt, perceived through vestibular signals and/or proprioceptive signals from the neck, without similarly compensating for the reflexive rotation of the eyes in the opposite direction.

Using a similar stimulus drawn down the forehead, we found an A-effect in the subjective tactile vertical (STV). To our knowledge, our study was the first to test the STV using *passive* tactile stimulation. Previous studies investigated the subjective *haptic* vertical (SHV) by asking participants to actively rotate a rod to align it with the direction of gravity (e.g. Bauermeister et al., 1964; Fraser et al., 2015; Guerraz et al., 2000; Hazlewood and Singer, 1969). SHV tasks involve multiple sensorimotor cues besides tactile inputs, such as efference copies of the motor commands (Wolpert and Ghahramani, 2000), proprioceptive signals from the arms and hands, and gravitational forces on those same body parts. All those signals could provide additional cues to the direction of gravity that would not contribute to the perception of a passive tactile stimulus on the forehead. Using a purely tactile stimulus, we found participants' STV was biased toward the longitudinal head axis (an A-effect). Since we spend most of our waking lives with our head upright on our shoulders, the brain may hold this default upright position as a strong ‘prior’ prediction of the orientation of the head with respect to the body (Alberts et al., 2016; Clemens et al., 2011; De Vrijer et al., 2008, 2009). When the head is tilted, noise is added to vestibular signals, likely because of the non-uniform distribution of hair cells on the otoliths (De Vrijer et al., 2008; Tarnutzer et al., 2009b). Within a Bayesian optimal cue integration framework, noisy sensory cues should contribute less to an overall percept than precise cues, because of their unreliability (Ernst and Banks, 2002; Ernst and Bülthoff, 2004). As vestibular signals became less reliable with the head tilted, perception of the STV may have been increasingly dominated by an upright head prior, leading to an A-effect.

**Fig. 4:**
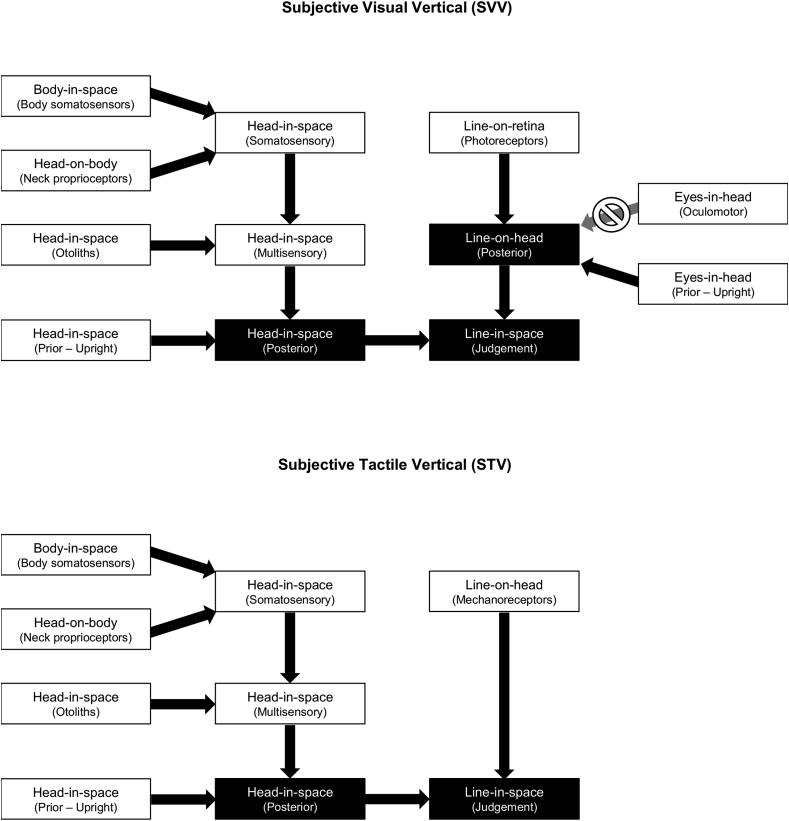
Proposed models of subjective visual verticality (SVV) perception (top) and subjective tactile verticality (STV) perception (bottom), adapted from the SVV model by Clemens et al. (2011). Multisensory cues are weighted according to their reliability and combined with Bayesian prior predictions that the head is upright in space and, in the case of SVV, that the eyes are upright within the head. Unlike Clemens et al., 2011, we propose that oculomotor ‘eyes-in-head’ cues are not taken into account in the SVV, resulting in over-compensation for head tilts (i.e. an E-effect). Because tilting the head increases vestibular noise, the upright head prior dominates in STV judgements and leads to under-compensation for head tilts (i.e. an A-effect).

Our results suggest that the brain uses surprisingly similar processes for judging the verticality of visual and passive tactile stimuli. Based on our findings and previous related studies, we propose adapted models of visual and tactile verticality perception in Fig. 4. In both cases, vestibular and proprioceptive signals are integrated with ‘line-on-retina’ (SVV) or ‘line-on-head’ (STV) cues and an upright head prior. As the head is tilted, the vestibular signals become noisier, so they are given less weight in combination with the prior and other sensory cues. The head is thus perceived as tilted with respect to the body, but the degree of tilt is underestimated. In the case of passive tactile stimulation of the forehead (Fig. 4, bottom), the brain therefore under-compensates for the full degree of head tilt, resulting in a STV biased toward the longitudinal head axis (but not completely aligned with it). In the case of visual stimulation (Fig. 4, top), the brain fails to adequately integrate an additional relevant cue—the position of the eyes within the head—which is already providing some mechanical compensation for the head tilt due to the ocular counter-roll reflex. This leads to an over-compensation for the head tilt, and a SVV biased in the opposite direction.

The idea that vestibular signals degrade as the head is tilted is supported by our finding that the precision of verticality judgements decreased in the rightward head position, relative to the upright head position. This reduction in precision was especially pronounced for ballet dancers’ judgements of tactile stimulus direction. Given the extensive proprioceptive and vestibular training that ballet dancers receive, we had predicted that their verticality judgements would be less affected than non-experts by tilted head postures. Other studies have shown that professional dancers have better balance and proprioceptive abilities than amateur dancers and non-dancers (Chatfield et al., 2007; Crotts et al., 1996; Golomer et al., 1999; Jola et al., 2011; Ramsay and Riddoch, 2001; Rein et al., 2011). Such bodily expertise may be limited to the kinds of movements and postures the dancers typically use in their routines. As such, their training might not generalise to other movements such as a simple head tilt. Nevertheless, this would not explain why precision was more dramatically reduced by head tilt in dancers than non-dancers.

On the other hand, if ballet dancers were particularly reliant on vestibular signals to judge the orientation of their body relative to the direction of gravity, then they might be especially affected by manipulations such as head tilts that add noise to those sensory inputs. Our results therefore suggest that ballet dancers might weigh vestibular signals more heavily than non-dancers in their verticality judgements (c.f. Nigmatullina et al., 2015, for contrary evidence that ballet dancers suppress vestibular signals of yaw-plane rotations in vertigo perception). This potentially increased reliance on vestibular signals was dissociated from the precision of those signals, meaning that dancers' verticality judgements were noisier during head tilts. However, it is not clear why this impaired precision was particularly pronounced in dancers' *tactile* verticality judgements. One possible explanation could be that the dancers’ judgements of visual verticality tended to be less precise than their judgements of tactile verticality overall, although this trend was not statistically significant (*p* = .066). If they were already less sensitive to visual stimulus direction when upright, then there may have been less room for a further decrement in visual task performance. We stress, however, that these are only tentative suggestions to explain an unexpected pattern of results. Further research will be needed to determine the consequences of dance training for verticality perception.

Our experiment offered several methodological advantages that allow us to build upon previous studies. First, we used similar stimuli to test both the SVV and the STV, allowing direct comparisons between the visual and tactile modalities. Second, we eliminated any visual cues to the direction of gravity in the SVV condition, forcing participants to rely upon proprioceptive and vestibular signals to make their judgements about the direction of the visual stimulus. Third, we used passive tactile stimulation of the forehead in the STV condition, rather than active manipulation of a rod. This rules out additional cues to verticality from the motor system, proprioceptive signals from the arms and hands, and gravitational forces on the upper limbs.

Despite these notable strengths, our study does have some limitations. To reduce the study duration, we only compared rightward head tilts to an upright head condition. We did not test the effects of leftward head tilts, so we cannot rule out the possibility that any effects we observed are asymmetrical. Additionally, tilting the head simultaneously affects inputs from both the vestibular otolithic organs and proprioceptive neck afferents, so we cannot separate the contributions of those signals to visual and tactile verticality perception. Future research could, for example, use galvanic vestibular stimulation to isolate the contributions of vestibular signals to verticality perception in the visual and tactile modalities. Finally, we did not measure the ocular counter-roll reflex in our participants. Although our finding of an E-effect in the SVV task but not the STV task is consistent with an account based on ocular counter-roll, there may be other possible explanations. Future studies could directly measure the ocular counter-roll reflex to better determine its relation to the E-effect in visual verticality judgements.

To summarise, our findings suggest that both ballet dancers and non-dancers show similar visual and tactile verticality perception, although the dancers showed a greater loss of precision in their tactile verticality judgements when tilting the head 30° rightward. Both groups showed a bias of the SVV against the direction of the head tilt (an E-effect) and a bias of the STV toward the direction of the head tilt (an A-effect). Despite these apparently opposing effects in the visual and tactile modalities, we have shown how a common Bayesian framework of verticality perception could account for both effects. Overall, this supports the idea of a Bayesian multisensory cue integration model of verticality perception that—in the absence of visual cues to the gravitational vertical—is unaffected by the sensory modality of the comparison stimulus, and only minimally affected by dance expertise.

## CRediT authorship contribution statement

**Brianna Beck:** Conceptualization, Methodology, Software, Formal analysis, Data curation, Writing - original draft, Writing - review & editing, Visualization, Project administration. **Alkistis Saramandi:** Conceptualization, Methodology, Formal analysis, Investigation, Writing - original draft, Writing - review & editing, Visualization. **Elisa Raffaella Ferrè:** Conceptualization, Methodology, Writing - review & editing. **Patrick Haggard:** Conceptualization, Methodology, Resources, Writing - review & editing, Supervision, Funding acquisition.

## References

[bib1] Alberts B.B.G.T., Selen L.P.J., Bertolini G., Straumann D., Medendorp W.P., Tarnutzer A.A. (2016). Dissociating vestibular and somatosensory contributions to spatial orientation. J. Neurophysiol..

[bib2] Alberts B.B.G.T., Selen L.P.J., Verhagen W.I.M., Medendorp W.P. (2015). Sensory substitution in bilateral vestibular a-reflexic patients. Phys. Rep..

[bib3] Aubert H. (1861). Eine scheinbare bedeutende Drehung von Objecten bei Neigung des Kopfes nach rechts oder links. Arch. für Pathol. Anat. Physiol. für Klin. Med..

[bib4] Barra J., Marquer A., Joassin R., Reymond C., Metge L., Chauvineau V., Pérennou D. (2010). Humans use internal models to construct and update a sense of verticality. Brain.

[bib5] Bauermeister M., Werner H., Wapner S. (1964). The effect of body tilt on tactual-kinesthetic perception of verticality. Am. J. Psychol..

[bib6] Betts G.A., Curthoys I.S. (1998). Visually perceived vertical and visually perceived horizontal are not orthogonal. Vis. Res..

[bib7] Bronstein A.M. (1999). The interaction of otolith and proprioceptive information in the perception of verticality: the effects of labyrinthine and CNS disease. Ann. N. Y. Acad. Sci..

[bib8] Ceyte H., Cian C., Trousselard M., Barraud P.-A. (2009). Influence of perceived egocentric coordinates on the subjective visual vertical. Neurosci. Lett..

[bib9] Chatfield S.J., Krasnow D.H., Herman A., Blessing G. (2007). A descriptive analysis of kinematic and electromyographic relationships of the core during forward stepping in beginning and expert dancers. J. Dance Med. Sci..

[bib10] Clemens I.A.H., De Vrijer M., Selen L.P.J., Van Gisbergen J.A.M., Medendorp W.P. (2011). Multisensory processing in spatial orientation: an inverse probabilistic approach. J. Neurosci..

[bib11] Crotts D., Thompson B., Nahom M., Ryan S., Newton R.A. (1996). Balance abilities of professional dancers on select balance tests. J. Orthop. Sports Phys. Ther..

[bib12] Curthoys I.S. (1996). The role of ocular torsion in visual measures of vestibular function. Brain Res. Bull..

[bib13] Day B.L., Fitzpatrick R.C. (2005). The vestibular system. Curr. Biol..

[bib14] Day R.H., Wade N.J. (1969). Mechanisms involved in visual orientation constancy. Psychol. Bull..

[bib15] De Vrijer M., Medendorp W.P., Van Gisbergen J.A.M. (2008). Shared computational mechanism for tilt compensation accounts for biased verticality percepts in motion and pattern vision. J. Neurophysiol..

[bib16] De Vrijer M., Medendorp W.P., Van Gisbergen J.A.M. (2009). Accuracy-precision trade-off in visual orientation constancy. J. Vis..

[bib17] Dichgans J., Diener H.C., Brandt T. (1974). Optokinetic-graviceptive interaction in different head positions. Acta Otolaryng.

[bib18] Dichgans J., Held R., Young L.R., Brandt T. (1972). Moving visual scenes influence the apparent direction of gravity. Science.

[bib19] Ernst M.O., Banks M.S. (2002). Humans integrate visual and haptic information in a statistically optimal fashion. Nature.

[bib20] Ernst M.O., Bülthoff H.H. (2004). Merging the senses into a robust percept. Trends Cognit. Sci..

[bib21] Faul F., Erdfelder E., Lang A.-G., Buchner A. (2007). G*Power 3: a flexible statistical power analysis program for the social, behavioral, and biomedical sciences. Behav. Res. Methods.

[bib22] Fraser L.E., Makooie B., Harris L.R. (2015). The subjective visual vertical and the subjective haptic vertical access different gravity estimates. PloS One.

[bib23] Golomer E., Crémieux J., Dupui P., Isableu B., Ohlmann T. (1999). Visual contribution to self-induced body sway frequencies and visual perception of male professional dancers. Neurosci. Lett..

[bib24] Golomer E., Dupui P. (2000). Spectral analysis of adult dancers' sways: sex and interaction vision-proprioception. Int. J. Neurosci..

[bib25] Groberg D.H., Dustman R.E., Beck E.C. (1969). The effect of body and head tilt in the perception of vertical: comparison of body and head tilt with left and right handed, male and female subjects. Neuropsychologia.

[bib26] Guerraz M., Luyat M., Poquin D., Ohlmann T. (2000). The role of neck afferents in subjective orientation in the visual and tactile sensory modalities. Acta Otolaryngol..

[bib27] Guerraz M., Poquin D., Luyat M., Ohlmann T. (1998). Head orientation involvement in assessment of the subjective vertical during whole body tilt. Percept. Mot. Skills.

[bib28] Hazlewood V., Singer G. (1969). Kinesthetic orientation judgments during lateral head, body and trunk tilt. Percept. Psychophys..

[bib29] Held R., Dichgans J., Bauer J. (1975). Characteristics of moving visual scenes influencing spatial orientation. Vis. Res..

[bib30] Hughes P.C., Brecher G.A., Fishkin S.M. (1972). Effects of rotating backgrounds upon the perception of verticality. Percept. Psychophys..

[bib53] Jola C., Davis A., Haggard P. (2011). Proprioceptive integration and body representation: insights into dancers’ expertise. Exp. Brain Res..

[bib32] Kingdom F.A.A., Prins N. (2016). Psychophysics: A Practical Introduction.

[bib33] MacNeilage P.R., Banks M.S., Berger D.R., Bülthoff H.H. (2007). A Bayesian model of the disambiguation of gravitoinertial force by visual cues. Exp. Brain Res..

[bib34] Mittelstaedt H. (1983). A new solution to the problem of the subjective vertical. Naturwissenschaften.

[bib35] Mittelstaedt H. (1997). Interaction of eye-, head-, and trunk-bound information in spatial perception and control. J. Vestib. Res..

[bib36] Müller G.E. (1916). Über das Aubertsche phänomen. Z. Sinnesphysiol..

[bib37] Nigmatullina Y., Hellyer P.J., Nachev P., Sharp D.J., Seemungal B.M. (2015). The neuroanatomical correlates of training – related perceptuo-reflex uncoupling in dancers. Cerebr. Cortex.

[bib38] Prins N., Kingdom F.A.A. (2018). Applying the model-comparison approach to test specific research hypotheses in psychophysical research using the Palamedes toolbox. Front. Psychol..

[bib39] Ramsay J.R.E., Riddoch M.J. (2001). Position-matching in the upper limb: professional ballet dancers perform with outstanding accuracy. Clin. Rehabil..

[bib40] Rein S., Fabian T., Zwipp H., Rammelt S., Weindel S. (2011). Postural control and functional ankle stability in professional and amateur dancers. Clin. Neurophysiol..

[bib41] Tarnutzer A.A., Bockisch C.J., Straumann D. (2010). Roll-dependent modulation of the subjective visual vertical: contributions of head- and trunk-based signals. J. Neurophysiol..

[bib42] Tarnutzer A.A., Bockisch C.J., Straumann D. (2009). Head roll dependent variability of subjective visual vertical and ocular counterroll. Exp. Brain Res..

[bib43] Tarnutzer A.A., Bockisch C., Straumann D., Olasagasti I. (2009). Gravity dependence of subjective visual vertical variability. J. Neurophysiol..

[bib44] Van Beuzekom A.D., Van Gisbergen J.A.M. (2000). Properties of the internal representation of gravity inferred from spatial direction and body-tilt estimates. J. Neurophysiol..

[bib45] Wade N.J. (1968). Visual orientation during and after lateral head, body, and trunk tilt. Percept. Psychophys..

[bib46] Wade N.J. (1969). The effect of stimulus line variations on visual orientation with head upright and tilted. Aust. J. Psychol..

[bib47] Wade S.W., Curthoys I.S. (1997). The effect of ocular torsional position on perception of the roll-tilt of visual stimuli. Vis. Res..

[bib48] Winnick A., Sadeghpou S., Sova M., Otero-Millan J., Kheradmand A. (2019). No handedness effect on spatial orientation or ocular counter-roll during lateral head tilts. Phys. Rep..

[bib49] Witkin H.A., Asch S.E. (1948). Studies in space orientation. IV. Further experiments on perception of the upright with displaced visual fields. J. Exp. Psychol..

[bib52] Wolpert D.M., Ghahramani Z. (2000). Computational principles of movement neuroscience. Nat. Neurosci..

[bib51] Zupan L.H., Merfeld D.M. (2003). Neural processing of gravito-inertial cues in humans. IV. Influence of visual rotational cues during roll optokinetic stimuli. J. Neurophysiol..

